# Population structure in the native range predicts the spread of introduced marine species

**DOI:** 10.1098/rspb.2013.0409

**Published:** 2013-06-07

**Authors:** Michelle R. Gaither, Brian W. Bowen, Robert J. Toonen

**Affiliations:** 1 Section of Ichthyology, California Academy of Sciences, 55 Music Concourse Drive, San Francisco, CA 94118, USA; 2 Hawai‘i Institute of Marine Biology, University of Hawai‘i at Mānoa, PO Box 1346, Kāne‘ohe, HI 96744, USA

**Keywords:** alien species, invasiveness, introduction success, meta-analysis, genetic structure

## Abstract

Forecasting invasion success remains a fundamental challenge in invasion biology. The effort to identify universal characteristics that predict which species become invasive has faltered in part because of the diversity of taxa and systems considered. Here, we use an alternative approach focused on the spread stage of invasions. *F*_ST_, a measure of alternative fixation of alleles, is a common proxy for realized dispersal among natural populations, summarizing the combined influences of life history, behaviour, habitat requirements, population size, history and ecology. We test the hypothesis that population structure in the native range (*F*_ST_) is negatively correlated with the geographical extent of spread of marine species in an introduced range. An analysis of the available data (29 species, nine phyla) revealed a significant negative correlation (*R*^2^ = 0.245–0.464) between *F*_ST_ and the extent of spread of non-native species. Mode *F*_ST_ among pairwise comparisons between populations in the native range demonstrated the highest predictive power (*R*^2^ = 0.464, *p* < 0.001). There was significant improvement when marker type was considered, with mtDNA datasets providing the strongest relationship (*n* = 21, *R*^2^ = 0.333–0.516). This study shows that *F*_ST_ can be used to make qualitative predictions concerning the geographical extent to which a non-native marine species will spread once established in a new area.

## 1. Introduction

The rate of species introductions has increased dramatically in modern times correlating with human population growth, increased international trade and advances in transportation [1–3]. While most introduced species never become established, those that persist can have serious economic impacts [4,5], consequences to human health [6] and pose a threat to biodiversity and ecosystem function [7–9]. Wilcove *et al.* [10] estimated that 42 per cent of endangered species in the United States are under direct threat from invasive species: an estimate that is much higher in rare taxa [11]. Consequently, biological invasions are regarded as one of the greatest modern threats to global biodiversity [12].

Forecasting which species will become invasive and which ecosystems are most vulnerable is of great scientific and practical interest [3]. Despite considerable efforts, the identification of universal characteristics that predict the success of invasive species remains elusive [13,14]. However, failure to find generalized predictive traits is not surprising given the diversity of taxa and ecosystems subject to invasion. Furthermore, characteristics important to invasion success are likely to vary among the different stages of invasion. The accumulating evidence indicates that taxon specific traits such as reproductive strategy, growth rate, environmental tolerances and diet specificity [13,15,16], combine with introduction dynamics such as habitat match and propagule pressure [17–19], to produce successful invaders [13,14]. Distilling this complexity down to even a few metrics that predict invasion success across taxa would be valuable to ecologists and managers working to control introduced species. One possible metric is *F*_ST_, a common measure of population structure based on the alternate fixation of alleles between populations (reviewed by Holsinger & Weir [20] and Bird *et al*. [21]). Because only individuals that survive dispersal events, find suitable habitat and successfully reproduce are contributing to population gene pools, *F*_ST_ is a potential proxy for realized dispersal (but see [22–24]). While *F*-statistics have been used to estimate genetic differentiation and to infer migration rates among native populations (i.e. [25]), they have not previously been used to predict the outcome of introduction events.

### (a) The role of *F*_ST_ in predicting invasion success: a Hawaiian case study

**Figure 1.**
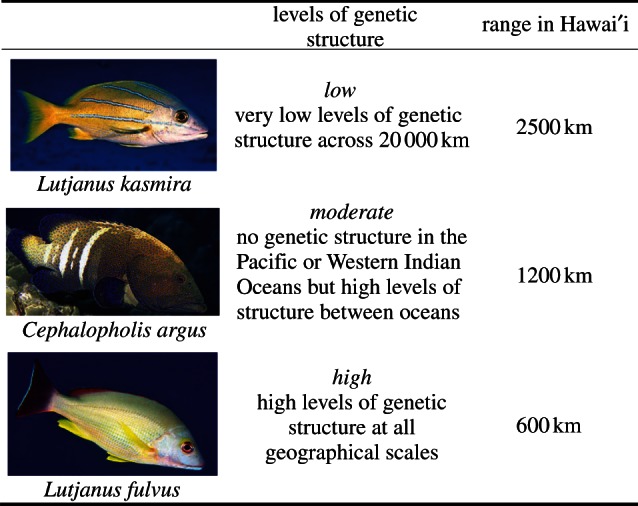
Genetic structure in the native range for each of the three fishes introduced to Hawai‘i. Level of genetic structure is described and the extent of spread within the introduced range is provided.

The relationship between *F*_ST_ and invasion success first came to our attention while studying introduced fishes in Hawai‘i. During the 1950s three fishes: the bluestripe snapper, *Lutjanus kasmira*, the blacktail snapper, *Lutjanus fulvus* and the peacock hind, *Cephalopholis argus* were deliberately introduced into Hawaiian waters [26–28]. These three species were introduced during the same time period and in roughly equal numbers (*n* = 2204–3175), yet they demonstrated contrasting patterns of success [28]. *Lutjanus kasmira*, with the widest distribution in Hawai‘i, demonstrates little genetic structure (low *F*_ST_) across nearly 20 000 km of its natural range (figure 1; [29,30]). By contrast, *L. fulvus,* with the smallest Hawaiian distribution of the three species, showed significant population structure at all geographical scales [30]. *Cephalopholis argus* demonstrated an intermediate pattern [31]. Given the myriad of factors that influence invader success, we were surprised by the relationship between population structure in the native range and the extent of spread demonstrated by these introduced fishes. This finding prompted the question of whether this relationship is broadly applicable to marine invaders. Here, we present an analysis of the available data, across 29 species and nine phyla, to determine whether there is a significant correlation between *F*_ST_, as a summary statistic surrogate for realized dispersal, and the extent of spread (invasiveness) in introduced marine species.

## 2. Material and methods

### (a) Literature search

We conducted a Web of Science search using the following Boolean combination (phylogeography, genetic, molecular), and (invasive, introduced, alien species) to identify invasive species for which genetic surveys in the native range have been conducted. The initial literature search was conducted in August 2011. The search resulted in 125 species of potential interest (see the electronic supplementary material, table S1). For each of these 125 taxa, an additional search based on the species scientific name was conducted. For inclusion in our study, we set the following criteria: (i) the species must be primarily marine or estuarine (diadromous species were not considered), (ii) a genetic survey of at least three native locations must be available, and (iii) the extent of spread in the introduced region must be documented. The majority of species were omitted from consideration owing to the lack of published population genetic data. Suspected species complexes and species with ambiguous native ranges were omitted from the dataset (i.e. *Botryllus schlosseri*, *Ciona intestinalis* and *Styela clava*). For each case study, we recorded marker type and global and pairwise *F*_ST_ values as reported in the published literature. Because demographic data are rarely available for marine species we recorded expected heterozygosity (*H*_E_) as a proxy for native population size. In studies where Mantel tests were conducted to test for isolation by distance (IBD) the slope of the regression was recorded. The extent of native range surveyed (km) was calculated as the shortest straight-line overwater distance between the two most distant sample sites. If desired *F*_ST_ values were not reported, haplotype/allele frequency files were created from the data published in tables in conjunction with sequences downloaded from GenBank (http://www.ncbi.nlm.nih.gov/genbank/). When sufficient data were not available, authors were contacted and the missing data were requested. From these reconstructed datasets, *H*_E_ was calculated and global and pairwise *F*_ST_ [32] values between sample locations were estimated using the ‘compute pairwise differences’ option and 20 000 permutations in arlequin [33].

In cases such as the European green crab, *Carcinus maenas*, where multiple surveys of the native range were conducted using the same genetic marker [34,35], we used the study that reported the most complete dataset and/or covered the greatest geographical area (for *C. maenas* data from Darling *et al*. [34]). For two species (*C. maenas* and the sea walnut, *Mnemiopsis leidyi*), data from two marker types were included (see the electronic supplementary material, table S2). Several species have been introduced to multiple regions around the globe (i.e. *Microcosmus squamiger*, *Caprella mutica*, *Nematostella vectensism* and *Mya arenaria*). In these cases, we evaluated the extent of spread in the region with the earliest date of detection. The edible alga wakame (*Undaria pinnatifida*) is cultured in its native Asian range and has since been spread around the globe. We analysed the introduction to Argentina because this was the earliest introduction harbouring haplotypes from natural populations in the native range.

### (b) Defining the extent of spread

The maximum extent of spread (MES) in the introduced range was defined as the shortest straight-line overwater distance (km) between the two furthest points in the introduced range. This is a highly conservative estimate of range expansion and does not account for multiple introduction events, secondary introductions or human-mediated dispersal within the introduced range. Disjunct distributions of alien species are common and likely reflect multiple or secondary introductions. To account for multiple introductions, we measured a second metric: the continuous extent of spread (CES). CES is defined as the geographical distance over which there is suitable habitat and no gaps in distribution of greater than 100 km. For example, the Caribbean barnacle *Chthamalus proteus*, was first recorded on the Hawaiian island of O‘ahu in 1995. This species has since been recorded throughout the Main Hawaiian Islands and at Midway Atoll over 2000 km northwest of O‘ahu. *Chthamalus proteus* has not been detected at intermediate locations within the archipelago despite suitable habitat. MES for this species was, therefore, recorded as 2489 km (the distance between Hawai‘i Island and Midway Atoll), whereas CES is recorded as 529 km (the distance between the islands of Hawai‘i and Kaua‘i) to reflect the possibility that *C. proteus* was secondarily transported to Midway Atoll on the hull of a boat or on fishing gear, rather than naturally jumping the intervening 1960 km.

### (c) Relationship between *F*_ST_ and extent of spread

Using the pairwise *F*_ST_ values between populations in the native range, we calculated mean, median and mode values. In preliminary analyses, minimum and maximum *F*_ST_ were considered but were later omitted because these measures of genetic structure are heavily influenced by sampling scale and do not correlate well with other measures of *F*_ST_. All data were log-transformed prior to analyses including *F*_ST_ values (global, mean, median and mode) and geographical distances (km). Because values of *F*_ST_ can be zero or negative, we added 1 to each value prior to transformation [ln(*F*_ST_ + 1)]. Likewise, because the geographical scale over which genetic surveys are conducted (distance between two most distant sample sites) can influence the magnitude of genetic structure, we standardized global *F*_ST_ values by calculating *F*_ST_ per kilometre [ln(global *F*_ST_ + 1)/ln(km)]. We used ordinary least-squares (OLS) regression analyses to determine if *F*_ST_ values (global *F*_ST_, global *F*_ST_/km, and mean, median and mode *F*_ST_ values for the pairwise comparisons) are a good predictor of the extent of spread in the introduced range. Simulation studies indicate that OLS regression analyses are preferred if the purpose of the study is not to estimate the parameters of a functional relationship, but instead to simply forecast values of the response variable for given explanatory variables (reviewed in [36]). Genetic structuring of marine taxa is often correlated with geographical distance (IBD). Therefore, in the subset of the studies in which IBD statistics were reported, we tested for a correlation between the IBD slope and extent of spread. To evaluate the influence of alternative variables on invasive success, we used the generalized linear model (GLM) with extent of spread as the response variable, *F*_ST_ as the explanatory variable, and marker type and *H*_E_ (of native populations) as covariates. Statistical analyses were conducted using SPSS v. 17.0 (IBM, Armonk, New York) except the GLM that was calculated using JMP Pro v. 10.0 (SAS, Cary, North Carolina). Plots of observed versus predicted residuals did not reveal any patterns that would bias the results or interpretation of the regression model (data not shown).

## 3. Results

### (a) Description of the dataset

Searching the literature resulted in 32 cases across 29 species that met our selection criteria: 10 molluscs, four fishes, four crustaceans, three tunicates, two vascular plants and one each of cnidarian, echinoderm, ctenophore, annelid, sponge and an alga (see the electronic supplementary material, table S2). The majority of the studies were based on mitochondrial DNA (21 of 32), including cytochrome oxidase I (*n* = 16; one of these is a concatenated COI/ND2 dataset), cytochrome *b* (*n* = 3), control region (*n* = 1) and intergenic spacers (*n* = 1). Nuclear markers were used in eleven studies and included: microsatellites (*n* = 6), allozymes (*n* = 2), internal transcribed spacers (*n* = 2) and amplified fragment length polymorphism (*n* = 1). Species included in this study are native to regions across the globe including Europe, Asia, America, Australia, South America and Oceania. Introduced regions were also geographically diverse, and included the above continents plus southern Africa. The recording of the green crab, *C. maenas*, in North America in 1817 makes this the earliest introduction in the dataset, whereas the most recent event involved the detection of the sponge *Crambe crambe* in the Canary Islands in 1995. The number of native populations surveyed per species ranged from three to 33 (mean = 9.66). Sample sizes per location within the 29 studies ranged from nine to 70 individuals (mean = 25.69) (see the electronic supplementary material, table S2).

Genetic surveys were conducted over a wide range of geographical distances. The shortest distance was 50 km between five populations of the Japanese oyster drill, *Ocinebrellus inornatus*. The largest native range surveyed was for the bluestripe snapper, *L. kasmira*, for which 10 populations were sampled across nearly 20 000 km. Global *F*_ST_ values were obtained for 26 of 32 studies and ranged from 0 for the soft-shelled clam *M. arenaria* to 0.906 for the alga *U. pinnatifida*. After standardizing for the geographical range over which the genetic survey was conducted, *F*_ST_/km ranged from 0 to 0.002 with the starlet sea anemone *N. vectensism* demonstrating the highest value. Pairwise *F*_ST_ values were obtained for 30 of 32 studies. From these pairwise values we calculated mean *F*_ST_ (range = 0.002–0.943), median (range = −0.018 to 0.969) and mode (range = −0.100 to 1.00) (see the electronic supplementary material, table S2). In four studies, although pairwise *F*_ST_ values were available, the number of populations surveyed was low (3 or 4) and precluded the calculation of mode *F*_ST_. MES varied widely among species ranging from 42 km for the tunicate *Pyura praeputialis* to 4344 and 4497 km for the ctenophore *M. leidyi* and the bivalve *M. arenaria*, respectively. To take secondary introductions into account, we calculated CES for each species which resulted in a narrower range from 8 km for the alga *U. pinnatifida* to 2583 km for the bluestripe snapper *L. kasmira*.

### (b) Relationship between *F*_ST_ and extent of spread

Weersing & Toonen [37] found that global *F*_ST_ was poorly correlated with geographical study scale in marine organisms, explaining only 2 per cent of the variance among 149 studies. Likewise, we found no correlation between global *F*_ST_ and study scale in our smaller dataset (*n* = 30, *R*^2^ = 0.004, *p* = 0.754): a finding that may result from the inclusion of different marker types [38]. Similar to other studies, we found that correcting for geographical scale resulted in a slightly higher correlation between *F*_ST_ and the extent of spread (Global *F*_ST_/km; table 1; [38,39]). Contrary to expectations, we found no correlation between *F*_ST_ and *H*_E_.

**Table 1.** *F*_ST_ as a predictor of extent of spread of introduced species. Number of studies (*n*), regression coefficient (*R*^2^), and corresponding goodness of fit *F*-statistic and *p*-values are reported. *R*^2^ values in italics are significant (*α* = 0.05). Regression equations are reported for significant correlations. MES, maximum extent of spread; and CES, continuous extent of spread.

	all markers					mtDNA				
genetic structure	*n*	*R*^2^	goodness of fit *F*-statistic	*p-*value	regression equation	*n*	*R*^2^	goodness of fit *F*-statistic	*p-*value	regression equation
MES										
global *F*_ST_	26	0.011	0.255	0.618		19	0.036	0.635	0.436	
global *F*_ST_/km	26	0.017	0.422	0.522		19	0.045	0.804	0.382	
mean *F*_ST_	30	0.001	0.026	0.873		21	0.016	0.302	0.589	
median *F*_ST_	26	0.001	0.017	0.896		21	0.011	0.220	0.644	
mode *F*_ST_	26	0.001	0.032	0.859		19	0.010	0.169	0.686	
CES										
global *F*_ST_	26	*0.245*	7.801	0.010	*Y* = −2.62*x* + 6.68	19	*0.333*	8.497	0.010	*Y* = −3.29*x* + 7.13
global *F*_ST_/km	26	*0.288*	9.728	0.005	*Y* = −29.10*x* + 6.83	19	*0.392*	10.975	0.004	*Y* = −37.88*x* + 7.34
mean *F*_ST_	30	*0.294*	11.672	0.002	*Y* = −4.78*x* + 6.77	21	*0.387*	12.013	0.003	*Y* = −5.58*x* + 7.05
median *F*_ST_	26	*0.394*	18.201	<0.001	*Y* = −4.85*x* + 6.69	21	*0.492*	18.381	<0.001	*Y* = −5.28*x* + 6.83
mode *F*_ST_	26	*0.464*	20.814	<0.001	*Y* = −5.25*x* + 6.47	19	*0.516*	18.158	0.001	*Y* = −5.25*x* + 6.53

We found no correlation between genetic structure in the native range and MES. However, when we corrected for secondary introductions and possible human-mediated dispersal within the introduced range, we detected a significant negative correlation between genetic structure and CES. Regardless of the *F*_ST_ value used (global, mean, median or mode), we found significant correlations between population structure in the native range and CES (table 1). Global *F*_ST_ proved to be the weakest predictor of spread (*R*^2^ = 0.245, *p* = 0.010) with a slight improvement in predictive power when global *F*_ST_ was corrected for geographical scale of the study (global *F*_ST_/km, *R*^2^ = 0.288, *p* = 0.005). The best predictor of CES was the mode of the pairwise *F*_ST_ values (*R*^2^ = 0.464, *p* < 0.001). While not as strongly correlated with spread as mode *F*_ST_, both mean (*R*^2^ = 0.294, *p* = 0.002) and median (*R*^2^ = 0.394, *p* < 0.001) pairwise *F*_ST_ values were better predictors than global *F*_ST_.

**Figure 2.**
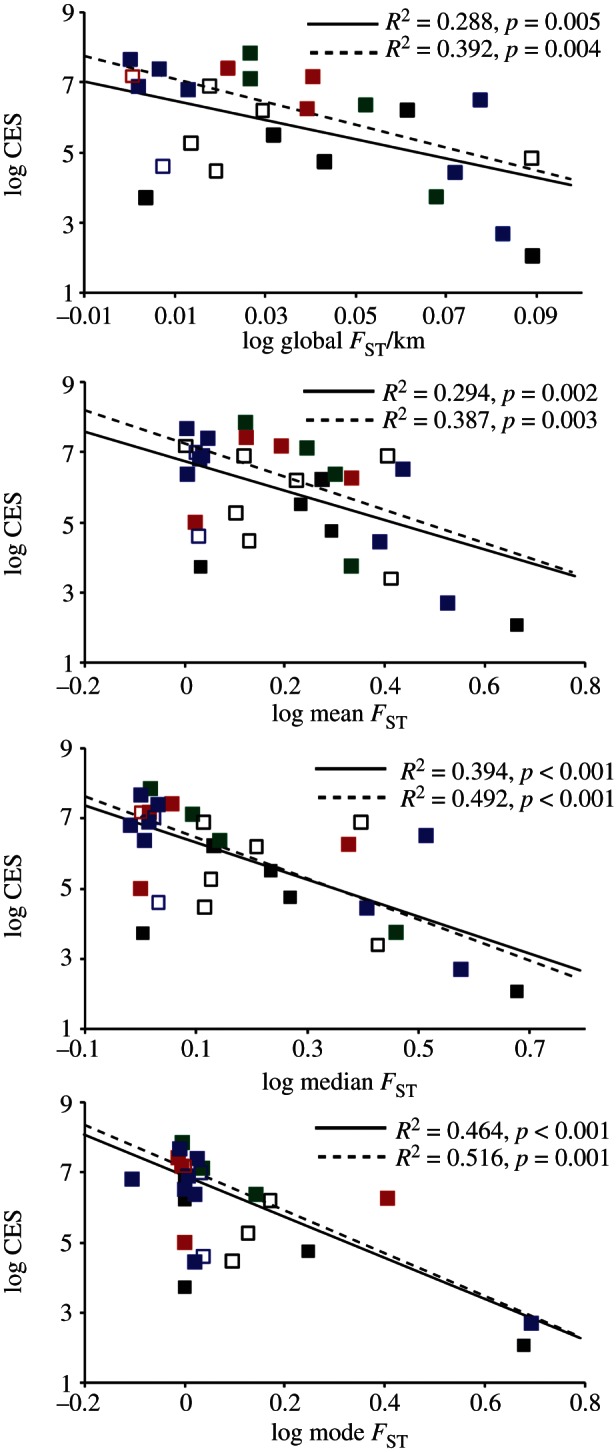
Population genetic structure (*F*_ST_) versus continuous extent of spread (CES) across nine marine phyla (molluscs, blue; crustaceans, red; fishes, green). Regression lines are plotted for the entire dataset (solid lines) and for just the mtDNA dataset (dashed lines). *R*^2^ values and corresponding *p*-values are shown.

We did not find a significant effect for marker type, taxon or *H*_E_ on the extent of spread (table 2). However, previous studies have shown that marker type is a significant covariate when modelling global *F*_ST_ [37–39]. For this reason, we ran regression analyses on the mitochondrial datasets (*n* = 21) to determine if the correlation between *F*_ST_ and extent of spread improved. In all cases correlations were stronger when analyses were restricted to the mtDNA datasets (table 1 and figure 2). The hierarchy of predictive power between the different measures of *F*_ST_ was consistent between the datasets with global *F*_ST_, providing the lowest predictive power (*R*^2^ = 0.333, *p* = 0.010) and the mode pairwise *F*_ST_ having the greatest predictive power (*R*^2^ = 0.516, *p* < 0.001).

**Table 2.** Results from generalized linear model used to evaluate the influence of alternative variables on invasive success. Extent of spread is the response variable, *F*_ST_ is the explanatory variable and marker type and *H*_E_ (of native populations) are covariates. *p*-values in italics are significant (*α* = 0.05).

factor	d.f.	*χ*^2^	*p*-value
CES			
marker type	1	0.075	0.784
global *F*_ST_/km	1	6.626	*0.010*
mean *F*_ST_	1	7.636	*0.006*
median *F*_ST_	1	11.607	*<0.001*
mode *F*_ST_	1	16.289	*<0.001*
* H*_E_	1	0.519	0.471

Tests for IBD among native populations were available for only 10 of the 32 studies. We found no significant correlation between IBD slope and MES (*R*^2^ = 0.044, *p* = 0.560). We detected a larger *R*^2^ value for the IBD slope versus CES comparison (*R*^2^ = 0.190; *p* = 0.208), however, the power to detect a significant correlation was limited owing to sample size.

## 4. Discussion

Identifying general characteristics that allow ecologists and managers to predict invaders has proved to be elusive. Here, we offer a metric with considerable power to predict the extent of spread in marine alien species. *F*_ST_, a measure of alternate fixation of alleles, is a common proxy for realized dispersal among natural populations. Our analysis reveals a strong negative correlation between genetic structure in the native range and the extent of spread of invasive marine species across a diversity of taxa (*R*^2^ = 0.245–0.464). An even stronger correlation was detected when marker type was taken into account (*R*^2^ = 0.333–0.516). While there are insufficient data to determine if *F*_ST_ can predict the success of initial invasions, our results indicate that this metric is suitable to qualitatively predict the extent of spread once an invasive species is established.

*F*_ST_ is not a simple measure of population differentiation, but instead is influenced by population size and genetic diversity (heterozygosity). There is a predicted relationship between population size, *H*_E_ and *F*_ST_ [40–42]. Under equilibrium conditions, large effective population sizes are expected to produce high genetic diversity, and low population structure (*F*_ST_). Consequently, our correlation between *F*_ST_ and invasive spread may indicate that species that sustain large genetically diverse populations make better invaders [43]. Direct population size estimates are not available for most marine species; however, if we assume drift/mutation equilibrium, heterozygosity can be a proxy for population size. We did not find the expected negative correlation between *F*_ST_ and *H*_E_ in our dataset nor was there a significant relationship between extent of spread and *H*_E_, indicating that native population size is not driving the correlations described here.

We restricted our study to marine species because of the strikingly different modes of dispersal between land and sea. Most marine organisms have a biphasic life cycle in which adults are benthic and largely sedentary. Dispersal, over even short distances (1–10 km), is achieved primarily during a pelagic phase (eggs and larvae); the length of which varies greatly among taxa, from 0 days to more than a year in a few taxa (spiny lobsters and eels). As a result, marine larvae can be transported 100's or even 1000's of km before settlement. However, the length of the pelagic larval phase is just one factor effecting dispersal in marine organisms. Timing of spawning, local oceanographic conditions, larval swimming and sensory ability and habitat requirements all play important roles in determining effective dispersal in marine organisms. These myriad of factors probably explain why a consistent correlation between the length of the pelagic phase and *F*_ST_ has not been found except over small geographical scales [37,39]. In the terrestrial realm, perhaps only wind dispersed seeds have a comparable mode of dispersal. Even in fresh water taxa, many of which also have pelagic larvae, dispersal is confined to the lakes and rivers of discrete drainage systems (i.e. zebra mussel in North America) that have no clear analogy in the sea.

### (a) *F*_ST_ as a proxy for realized dispersal

Because *F*_ST_ is a summary statistic, significant structure among populations may be a result of differences in effective population size, demographic or colonization history, migration, or some combination of these factors, especially for populations that may not have reached migration drift equilibrium, and thus direct interpretation of population structure in the context of gene flow can sometimes be problematic (reviewed by Hart & Marko [22], Lowe & Allendorf [23] and Marko & Hart [24]). We argue that the use of a summary statistic in this approach is warranted as it represents the cumulative effects of all factors on the genetic structure of the species, and despite the potential confounding variables, we find a significant relationship between *F*_ST_ and CES. Our conclusion, which is somewhat intuitive, is that species that show little or no population structure in their native range (e.g. effective dispersers, habitat or diet generalists, good competitors and broad environmental tolerances) also tend to become the most widespread invaders.

### (b) Natural realized dispersal and invasiveness

Our data indicate that up to half of the variation in the extent of spread of marine invasive species can be explained by native population structure as measured by *F*_ST_. Global *F*_ST_ had the lowest predictive power, explaining 25 per cent of the variation which increased to 39–46% using either median or mode *F*_ST_. While our dataset was not sufficient to test for the effect of marker type on estimates of genetic structure, measures of *F*_ST_ from mtDNA are generally higher than from nDNA, complicating direct comparisons of *F*_ST_ among marker types ([37–39], but see [44]). Here, we detected higher correlations across estimates of *F*_ST_ (with as much as 52% of the variance explained) when only the mtDNA datasets were considered.

While our findings provide a new measure of invasiveness, caution is indicated in the interpretation of *F*_ST_ values. Our review of the published literature resulted in a moderately sized dataset (29 species) of which the two data points with the highest *F*_ST_ values and correspondingly low CES had a large impact on the relationships revealed here (brown algae, *U. pinnatifida*; horn snail, *Batillaria attramentaria*). The former is anchored to substrate and the latter is believed to have a very brief pelagic larval stage. Omitting these two data points results in significant relationship only for median *F*_ST_ (*R*^2^ = 0.177, *p* = 0.026). Examining the data plots (figure 2), it becomes clear that much of the variation in the dataset is at moderate values of *F*_ST_ and CES, indicating that at these values *F*_ST_ has less predictive power. This leads us to conclude that *F*_ST_ can be used to make only qualitative predictions concerning the extent of spread of invasive species [45]. Future studies may provide additional data points to fill in the high end of the *F*_ST_ spectrum and clarify the pattern.

The amount of time that has elapsed since introduction will influence the extent of spread. In our dataset, there is a 12-fold difference in the number of years since the earliest and most recent introductions (the green crab, *C. maenas*, to North America in 1817 and the sponge *C. crambe* to the Canary Islands in 1995). However, correcting for this variation is not straightforward. First, the time disparity between first record of occurrence and the actual date of introduction can be vast. Some alien species go undetected for decades, and survey efforts vary considerably across geographical regions [3]. Second, rates of spread vary across taxa and even through time [46]. Therefore, we were not surprised to find no correlation between population structure and invasiveness when we corrected for time since introduction (extent of spread in km per year).

Given the numerous sources of variance in the dataset, the variety of factors that determine invasion success, and the diversity of taxa and systems examined, our finding that *F*_ST_ explains up to 52 per cent of the variance is remarkable. We suspect that the predictive power of *F*_ST_ would increase if analyses could be conducted at the level of individual phyla. However, there is a paucity of population genetic data on alien species. Of the 125 candidate species considered only 23 per cent had genetic data from the native range, and the taxonomic group with the greatest coverage (molluscs, *n* = 10) was insufficient for a robust analysis.

### (c) Continuous versus total extent of spread

The power of *F*_ST_ to predict the geographical spread of alien species is confounded by secondary introductions or human-mediated dispersal within the introduced range [34]. For example, fouling organisms such as tunicates, sponges and oysters can easily be translocated between harbours on boat hulls or fishing gear [47], whereas the larvae of some species can survive in the ballast water of ships [48–50]. The same mechanisms that promote long range introductions can also facilitate spread within the new range. The result is often disjunct distributions in the non-native range that circumvent suitable habitat: a scenario that is less likely with innate (natural) dispersal. Our data indicate that human-mediated secondary introductions are an important means of spread for many alien species.

### (d) Terrestrial and freshwater systems

Several reviews have attempted to identify characteristics that predict invader success. However, none have attempted to correlate realized dispersal with invasiveness. Kolar & Lodge [13] review the plant and animal literature, and examine 68 species-level characteristics many of which influence dispersal such as reproductive mode, dispersal mechanisms in plants, fecundity and length of juvenile period. Of those characteristics only reproductive mode in plants was predictive of invasive status (plants with vegetative reproduction were more likely to spread and become abundant). Hayes & Barry [14] examined a larger set of characteristics (115 across seven biological groups) and found that only climate/habitat match was significantly associated with exotic range size across biological groups but not across studies within groups. Here, we add a new quantifiable quality to the array of invasive characteristics.

### (e) Conclusions and applications

Using *F*-statistics to predict the outcome of marine introductions is a novel approach that shows considerable promise. *F*_ST_ as a surrogate for realized dispersal incorporates many of the species-level characteristics that are known to influence invader success: reproductive strategy, habitat specificity and ecology [13]. While our findings show that up to 52 per cent of the variance in the spread of marine invaders can be explained by values of *F*_ST_, our data do not address the important question of whether a species is likely to become established. Instead, *F*_ST_ would be most useful to wildlife managers when incorporated into specific risk assessment models with success and failure trees for each stage of introduction. In this context, *F_ST_* could be used to determine which species, once established, are likely to become widespread, providing wildlife officers with a stronger scientific foundation for setting management priorities.
